# Impact of Preoperative Time Intervals for Neoadjuvant Chemoradiotherapy on Short-term Postoperative Outcomes of Esophageal Cancer Surgery: A Population-based Study Using the Dutch Upper Gastrointestinal Cancer Audit (DUCA) Data

**DOI:** 10.1097/SLA.0000000000006476

**Published:** 2024-08-08

**Authors:** Jingpu Wang, Cas de Jongh, Zhouqiao Wu, Eline M. de Groot, Alexandre Challine, Sheraz R. Markar, Hylke J.F. Brenkman, Jelle P. Ruurda, Richard van Hillegersberg

**Affiliations:** * Department of Surgery, University Medical Center Utrecht, Utrecht, The Netherlands; † Key Laboratory of Carcinogenesis and Translational Research (Ministry of Education), Department of Gastrointestinal Surgery, Peking University Cancer Hospital and Institute, Beijing, China; ‡ Department of Digestive Surgery, AP-HP, Hôpital Saint Antoine, Paris, France; § Nuffield Department of Surgical Sciences, University of Oxford, Oxford, UK

**Keywords:** esophageal cancer, neoadjuvant chemoradiotherapy, short-term outcome, time interval, waiting time

## Abstract

**Objective:**

To clarify the impact of the preoperative time intervals on short-term postoperative and pathologic outcomes in patients with esophageal cancer who underwent neoadjuvant chemoradiotherapy (nCRT) followed by esophagectomy.

**Background:**

The impact of preoperative intervals on patients with esophageal cancer who received multimodality treatment remains unknown.

**Methods:**

Patients (cT1-4aN0-3M0) treated with nCRT plus esophagectomy were included using the Dutch national DUCA database. Multivariate logistic regression was used to determine the effect of different time intervals upon short-term postoperative and pathologic outcomes: diagnosis-to-nCRT intervals (≤5, 5–8, and 8–12 weeks), nCRT-to-surgery intervals (5–11, 11–17, and >17 weeks) and total preoperative intervals (≤16, 16–25, and >25 weeks).

**Results:**

Between 2010 and 2021, a total of 5052 patients were included. Compared with diagnosis-to-nCRT interval ≤5 weeks, the interval of 8 to 12 weeks was associated with a higher risk of overall complications (*P*=0.049). Compared with nCRT-to-surgery interval of 5 to 11 weeks, the longer intervals (11–17 and >17 weeks) were associated with a higher risk of overall complications (*P*=0.016; *P*<0.001) and anastomotic leakage (*P*=0.004; *P*=0.030), but the interval >17 weeks was associated with lower risk of ypN+ (*P*=0.021). The longer total preoperative intervals were not associated with the risk of 30-day mortality and complications compared with the interval ≤16 weeks, but the longer total preoperative interval (>25 weeks) was associated with higher ypT stage (*P*=0.010) and lower pathologic complete response rate (*P*=0.013).

**Conclusions:**

In patients with esophageal cancer undergoing nCRT and esophagectomy, prolonged preoperative time intervals may lead to higher morbidity and disease progression, and the causal relationship requires further confirmation.

Esophagectomy is the mainstay of curative treatment for patients with resectable esophageal cancer.^1–4^ Multimodality treatment, by adding neoadjuvant therapy (NAT) to surgery, leads to improved prognosis for locally advanced esophageal cancer when compared with surgery alone, and has therefore been widely adopted as the standard of care.^5–7^ The time interval from diagnosis to treatment is considered one of the main important indicators for evaluating the quality of cancer care.^8–23^ Patients with esophageal cancer undergoing NAT followed by surgery experience 2 waiting intervals before esophagectomy. The first interval occurs between diagnosis and NAT, while the second interval takes place between NAT and surgery. Several studies have attempted to determine the impact of these 2 intervals on the survival and pathologic results of patients with esophageal cancer receiving NAT plus surgery. Two previous Dutch studies suggested that a longer interval from diagnosis to the start of NAT had no significant effect on pT- /pN-status, R0 resection rate, and overall survival (OS) for patients with esophageal cancer who underwent radical esophagectomy.^14,21^ Several studies assessing the interval from NAT to esophagectomy have shown that a longer interval was associated with a higher pathologic complete response (pCR) rate.^24–28^ The preoperative intervals represent different aspects of the patient’s preoperative waiting; however, most of these studies only focus on a single category of preoperative intervals in the analysis.

Therefore, to comprehensively analyze the impact of the preoperative time intervals on short-term postoperative outcomes and pathologic results in patients with esophageal cancer who underwent NAT followed by esophagectomy, this national cohort study simultaneously included the diagnosis-to-NAT interval, NAT-to-surgery interval, and total preoperative interval (diagnosis to surgery). In the Netherlands, neoadjuvant chemoradiotherapy (nCRT) according to the CROSS-regimen plus surgery is recommended as a treatment for patients with resectable esophageal cancer (cT1b-4a N0-3 M0) since 2010.^29^ As a result, this study focuses exclusively on patients who received nCRT plus surgery.

## METHODS

This population-based cohort study was conducted using prospective data from the Dutch upper GI cancer audit (DUCA), a mandatory national auditing registry for all hospitals performing esophageal and gastric cancer surgery in the Netherlands since 2011.^30,31^ Data from every patient per participating hospital in the registry were verified on an individual level by specialized medical data managers to safeguard the data quality, perform validation checks, and minimize missing data. For this study, data included patient and tumor characteristics, treatment details, short-term postoperative outcomes (up to 30 days after surgery), and histopathologic results for esophageal cancer.^31,32^ The scientific committee of the Dutch Institute for Clinical Auditing (DICA) and DUCA approved this study, and no ethical approval or informed consent from patients was required in accordance with Dutch law.

### Patient Selection

All patients diagnosed with esophageal cancer in 2010–2021 were extracted from the DUCA database. The exclusion criteria consisted of patients with (1) recurrent tumors; (2) age <18 years old; (3) cT4b/cM1 tumors; (4) emergency surgery; (5) no curative esophagectomy or <80% planned cycles of NAT; (6) no date of diagnosis or the start of nCRT or surgery; (7) no complication data; and (8) endoscopic mucosal resection before esophagectomy.

### Variables

The variables extracted from the DUCA database include sex, age, Charlson comorbidity score, history of malignancy, history of thoracic or abdominal surgery, (body mass index, before surgery), weight loss (at diagnosis), year of diagnosis, referral from another hospital, histology, tumor location, cTNM stage, date of pathologic biopsy, start date of NAT, ASA score, type of resection, type of NAT, 80% completion of NAT, date of surgery, hospital volume, pTNM stage, R0 resection, postoperative complications, and 30-day mortality.

### Exposure

The exposure under investigation was diagnosis-to-nCRT interval (from diagnosis to the initiation of nCRT), nCRT-to-surgery interval (from the initiation of nCRT to surgery), and total preoperative interval (from diagnosis to surgery). The date of diagnosis was defined as the date of pathologic biopsy during upper gastrointestinal endoscopy. Based on the Dutch cancer care guideline, the targeted maximum time from the first outpatient visit to the start of treatment should be 6 weeks, and the interval can be extended by 3 weeks if the patient is referred to another hospital.^33,34^ Therefore, the diagnosis-to-nCRT time interval was categorized as ≤5 weeks, 5 to 8 weeks, and >8 weeks. Considering that the schedule of the CROSS nCRT is 5 weeks, this study categorized the nCRT-to-surgery interval as 5 to 11 weeks, so within the originally defined criteria in the CROSS trial, 11 to 17 weeks, and >17 weeks. The total preoperative time interval between diagnosis and surgery was categorized as ≤16 weeks, 16 to 25 weeks, and >25 weeks by adding both intervals together.

### Outcome Measures

The primary outcome measure was 30-day mortality, and secondary outcome measures included overall complications, anastomotic leakage, ypT stage, ypN stage, pCR (defined as ypT0N0), and R0 resection.

### Statistical Analysis

Clinical characteristics among the patients with different intervals were described by using frequencies and percentages, and χ^2^ and ANOVA tests were used to compare the clinical characteristics among them. Multiple imputation was used to impute missing values and generate 20 new data sets. Univariate and multivariate logistic regression analyses were used to compare the impact of different preoperative time intervals on short-term postoperative and pathologic outcomes. A 2-sided *P*<0.05 was considered statistically significant. All statistical analyses were performed by using SPSS version 25.0 software (SPSS).

## RESULTS

### Patient Population

**FIGURE 1:**
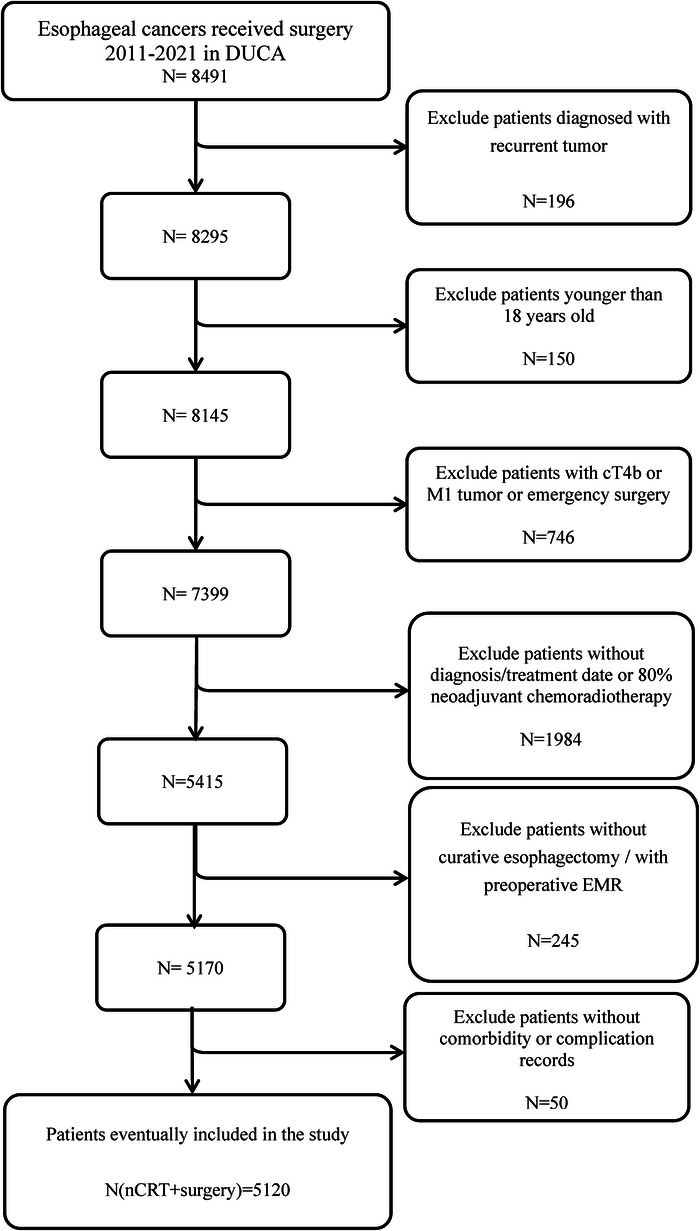
Selection process of patients. EMR indicates endoscopic mucosal resection.

A total of 8491 patients who underwent surgery between 2011 and 2021 were extracted, of whom 3371 patients were excluded, leaving 5120 included patients with resectable esophageal cancer who underwent nCRT plus curative esophagectomy (Fig. 1). Since the number of patients with diagnosis-to-nCRT interval >12 weeks was very limited (n=68; 1%), and these patients probably have serious risk of selection bias, they were also excluded, leaving 5052 patients included in the following analyses. The clinical characteristics of these patients are shown in Table 1.

**TABLE 1:** The Clinical Characteristics of Included Esophageal Cancer Patients

Variables	All (5052)
Age, n (%)	
18–64	2203 (43.6)
65–80	2733 (54.1)
≥80	116 (2.3)
BMI, n (%)	
<20	308 (6.1)
20–25	1874 (37.1)
25–30	2023 (40.0)
≥30	822 (16.3)
Unknown	25 (0.5)
Weight loss	
<5 kg	2525 (50.0)
≥5 kg	2133 (42.2)
Unknown	394 (7.8)
Sex, n (%)	
Male	3997 (79.1)
Female	1055 (20.9)
Charlson score, n (%)	
0	2449 (48.5)
1	1290 (25.5)
2+	1313 (26.0)
History of malignancy, n (%)	
No	4345 (86.0)
Yes	707 (14.0)
History of thoracic and abdominal surgery, n (%)	
No	3640 (72.1)
Yes	1406 (27.8)
Unknown	6 (0.1)
Referral, n (%)	
No	1166 (23.1)
Yes	3529 (69.9)
Unknown	357 (7.1)
Year of diagnosis, n (%)	
2010–2013	1233 (24.4)
2014–2017	2223 (44.0)
2018–2021	1596 (31.6)
Histology, n (%)	
Adenocarcinoma	4007 (79.3)
Squamous cell carcinoma	921 (18.2)
Other	79 (1.6)
Unknown	45 (0.9)
Tumor location, n (%)	
Intrathoracal proximal esophagus	51 (1.0)
Intrathoracal middle esophagus	579 (11.5)
Intrathoracal distal esophagus	3585 (71.0)
Esophagogastric junction	832 (16.5)
Unknown	5 (0.1)
cT stage, n (%)	
T1	36 (0.7)
T2	991 (19.6)
T3	3909 (77.4)
T4	116 (2.3)
cN stage, n (%)	
N0	1732 (34.3)
N1	2119 (41.9)
N2	983 (19.5)
N3	134 (2.7)
Unknown	84 (1.7)
ASA score, n (%)	
1	682 (13.5)
2	3096 (61.3)
3	1216 (24.1)
4	30 (0.6)
Unknown	28 (0.6)
Type of resection, n (%)	
Transhiatal esophagectomy	1075 (21.3)
Transthoracic esophagectomy	3977 (78.7)
Diagnosis-to-nCRT interval, wk, n (%)	
≤5	2474 (49.0)
5–8	2130 (42.2)
8–12	448 (8.9)
nCRT-to-surgery interval, wk, n (%)	
5–11	667 (13.2)
11–17	3424 (67.8)
>17	961 (19.0)
Total interval (diagnosis to surgery), wk, n (%)	
≤16	719 (14.2)
16–25	3780 (74.8)
>25	553 (10.9)
Hospital volume, n (%)	
<40	2061 (40.8)
≥40	2991 (59.2)

### Diagnosis-to-nCRT Interval

The diagnosis-to-nCRT interval was ≤5 weeks in 2474 (49%) patients, 5 to 8 weeks in 2130 (42%) patients, and 8 to 12 weeks in 448 (9%) patients.

The clinical characteristics of patients with esophageal cancer with different intervals are shown in Supplemental Table 1 (Supplemental Digital Content 1, http://links.lww.com/SLA/F243). Patients with longer intervals had older age, worse Charlson and ASA scores, more frequently a history of malignancy, more referrals, earlier year of diagnosis, earlier cT stage, shorter nCRT-to-surgery interval, and longer total preoperative interval.

Patients with intervals of ≤5 weeks, 5 to 8 weeks, and 8 to 12 weeks exhibited 30-day mortality rates of 2.5%, 3.1%, and 4.7%, with overall complication rates of 59.6%, 61.0%, and 65.8%, and anastomotic leak rates of 22.6%, 23.2%, and 26.7%, respectively. R0 resection rates were 96.2%, 95.7%, and 97.4%, with pCR rates at 20.3%, 18.4%, and 20.5%. The results of multivariate logistic regression analysis showed that compared with the interval ≤5 weeks, the interval of 8 to 12 weeks was independently associated with a higher risk of overall complications (OR: 1.27, 95% CI: 1.00–1.60, *P*=0.049), but the intervals of 5 to 8 weeks and 8 to 12 weeks were not independently associated with worse pathologic results (*P*>0.05) (Table 2).

**TABLE 2:** Comparison of the Effects of Different Diagnosis-to-nCRT Intervals on Short-term Outcomes and Pathologic Results

Diagnosis-to-nCRT interval	Univariate OR (95% CI)	*P*	Adjusted OR (95% CI)	*P*
30-day mortality (5052)				
≤5 wk (2474)	Reference			
5–8 wk (2130)	1.224 (0.862, 1.737)	0.258	1.097 (0.748, 1.607)	0.636
8–12 wk (448)	1.882 (1.136, 3.117)	**0.014**	1.306 (0.729, 2.339)	0.370
Overall complications (5052)				
≤5 wk (2474)	Reference			
5–8 wk (2130)	1.060 (0.942, 1.194)	0.331	1.050 (0.921, 1.197)	0.467
8–12 wk (448)	1.308 (1.059, 1.616)	**0.013**	1.265 (1.001, 1.598)	**0.049**
Anastomotic leakage (3877)				
≤5 wk (1886)	Reference			
5–8 wk (1650)	1.038 (0.886, 1.215)	0.647	1.046 (0.879, 1.244)	0.613
8–12 wk (341)	1.249 (0.960, 1.626)	0.098	1.215 (0.905, 1.632)	0.196
Non-R0 resection (4648)				
≤5 wk (2238)	Reference			
5–8 wk (1991)	1.144 (0.841, 1.556)	0.393	1.141 (0.811, 1.604)	0.449
8–12 wk (419)	0.691 (0.366, 1.308)	0.256	0.643 (0.323, 1.280)	0.208
Higher ypT stage (T0-2 vs T3-4) (4883)				
≤5 wk (2386)	Reference			
5–8 wk (2064)	1.039 (0.921, 1.172)	0.535	1.049 (0.917, 1.200)	0.486
8–12 wk (433)	1.064 (0.863, 1.310)	0.562	1.008 (0.797, 1.274)	0.948
Higher ypN stage (N0 vs. N+) (4895)				
≤5 wk (2391)	Reference			
5–8 wk (2070)	1.004 (0.890, 1.132)	0.949	1.038 (0.908, 1.187)	0.583
8–12 wk (434)	0.810 (0.654, 1.004)	0.054	0.823 (0.648, 1.046)	0.111
pCR (4887)				
≤5 wk (2388)	Reference			
5–8 wk (2065)	0.886 (0.763, 1.029)	0.112	0.884 (0.749, 1.045)	0.148
8–12 wk (434)	1.013 (0.786, 1.306)	0.919	1.129 (0.849, 1.502)	0.405

Bold values are statistical significance of *P* < 0.05.

The adjusted variables for 30-day mortality, overall complications, and anastomotic leakage included sex, age, Charlson score, history of malignancy, history of thoracic or abdominal surgery, BMI, weight loss, year at diagnosis, histology, tumor location, cT stage, cN stage, ASA, type of resection, preoperative total interval, nCRT-to-surgery interval, and hospital volume. The adjusted variables for non-R0 resection, higher ypT stage, higher ypN stage, and pCR included history of malignancy, year at diagnosis, histology, tumor location, cT stage, cN stage, total preoperative interval, nCRT-to-surgery interval, and hospital volume.

BMI indicates body mass index.

### nCRT-to-surgery Interval

The nCRT-to-surgery interval was 5 to 11 weeks in 667 (13%) patients, 11 to 17 weeks in 3424 (68%) patients, and >17 weeks in 961 (19%) patients.

The clinical characteristics of patients with esophageal cancer with different intervals are shown in Supplemental Table 2 (Supplemental Digital Content 1, http://links.lww.com/SLA/F243). Patients with longer intervals had older age, more weight loss, worse Charlson and ASA scores, more referrals, more recent year of diagnosis, more transthoracic esophagectomies, shorter diagnosis-to-nCRT interval, longer total preoperative interval, and higher hospital volume (≥40 esophagectomies annually).

Patients with intervals of 5 to 11 weeks, 11 to 17 weeks, and >17 weeks exhibited 30-day mortality rates of 1.9%, 2.8%, and 4.3%, with overall complication rates of 53.8%, 60.3%, and 67.0%, and anastomotic leak rates of 16.5%, 23.9%, and 26.7%, respectively. R0 resection rates were 96.3%, 95.9%, and 96.7%, with pCR rates at 19.1%, 20.2%, and 17.5%. The results of multivariate logistic regression analysis showed that compared with the interval of 5 to 11 weeks, the intervals of 11 to 17 weeks and >17 weeks were independently associated with a higher risk of overall complications (OR: 1.28, 95% CI: 1.04–1.56, *P*=0.016; OR: 1.70, 95% CI: 1.28–2.27, *P*<0.001) and anastomotic leakage (OR: 1.49, 95% CI: 1.13–1.96, *P*=0.004; OR: 1.53, 95% CI: 1.04–2.26, *P*=0.030), and the interval >17 weeks was independently associated with lower risk of ypN+ (OR: 0.71, 95% CI: 0.53–0.95, *P*=0.021) (Table 3).

**TABLE 3:** Comparison of the Effects of Different nCRT-to-surgery Intervals on Short-term Outcomes and Pathologic Results

nCRT-to-surgery interval	Univariate OR (95% CI)	*P*	Adjusted OR (95% CI)	*P*
30-day mortality (5052)				
5–11 wk (667)	Reference		Reference	
11–17 wk (3424)	1.451 (0.808, 2.606)	0.212	1.505 (0.773, 2.929)	0.229
>17 wk (961)	2.242 (1.192, 4.217)	**0.012**	1.580 (0.657, 3.801)	0.308
Overall complications (5052)				
5–11 wk (667)	Reference		Reference	
11–17 wk (3424)	1.304 (1.103, 1.540)	**0.002**	1.278 (1.047, 1.561)	**0.016**
>17 wk (961)	1.743 (1.423, 2.135)	**<0.001**	1.703 (1.278, 2.269)	**<0.001**
Anastomotic leakage (3877)				
5–11 wk (589)	Reference		Reference	
11–17 wk (2646)	1.592 (1.258, 2.014)	**<0.001**	1.491 (1.133, 1.963)	**0.004**
>17 wk (622)	1.846 (1.394, 2.446)	**<0.001**	1.533 (1.042, 2.256)	**0.030**
Non-R0 resection (4648)				
5–11 wk (656)	Reference		Reference	
11–17 wk (3178)	1.114 (0.715, 1.737)	0.633	1.166 (0.694, 1.957)	0.562
>17 wk (814)	0.903 (0.516, 1.581)	0.722	0.740 (0.330, 1.660)	0.465
Higher ypT stage (T0-2 vs T3-4) (4883)				
5–11 wk (642)	Reference		Reference	
11–17 wk (3308)	1.035 (0.870, 1.232)	0.695	0.959 (0.779, 1.182)	0.696
>17 wk (933)	1.211 (0.986, 1.486)	0.068	0.848 (0.633, 1.136)	0.269
Higher ypN stage (N0 vs N+) (4895)				
5–11 wk (643)	Reference		Reference	
11–17 wk (3320)	0.968 (0.814, 1.150)	0.709	0.912 (0.741, 1.122)	0.382
>17 wk (932)	0.845 (0.687, 1.039)	0.111	0.708 (0.528, 0.949)	**0.021**
pCR (4887)				
5–11 wk (643)	Reference		Reference	
11–17 wk (3312)	1.070 (0.864, 1.325)	0.535	1.154 (0.893, 1.492)	0.274
>17 wk (932)	0.897 (0.692, 1.162)	0.409	1.337 (0.935, 1.912)	0.111

Bold values are statistical significance of *P* < 0.05.

The adjusted variables for 30-day mortality, overall complications, and anastomotic leakage included sex, age, Charlson score, history of malignancy, history of thoracic or abdominal surgery, BMI, weight loss, year at diagnosis, histology, tumor location, cT stage, cN stage, ASA, type of resection, diagnosis-to-nCRT interval, total preoperative interval, and hospital volume. The adjusted variables for non-R0 resection, higher ypT stage, higher ypN stage, and pCR included history of malignancy, year at diagnosis, histology, tumor location, cT stage, cN stage, diagnosis-to-nCRT interval, total preoperative interval, and hospital volume.

BMI indicates body mass index.

### Total Preoperative Time Interval (Diagnosis to Surgery)

The total interval from diagnosis to surgery was ≤16 weeks in 719 (14%) patients, 16 to 25 weeks in 3780 (75%) patients, and >25 weeks in 553 (11%) patients.

The clinical characteristics of patients with esophageal cancer with different intervals are shown in Supplemental Table 3 (Supplemental Digital Content 1, http://links.lww.com/SLA/F243). Patients with longer total intervals had older age, worse Charlson score and ASA score, more frequently a history of malignancy and thoracic/abdominal surgery, more referrals, more recent years of diagnosis, more transthoracic esophagectomies, longer diagnosis-to-nCRT interval, longer nCRT-to-surgery interval, and higher hospital volume (≥40 esophagectomies annually).

Patients with intervals of ≤16 weeks, 16 to 25 weeks, and >25 weeks exhibited 30-day mortality rates of 2.1%, 2.8%, and 5.6%, with overall complication rates of 55.1%, 60.8%, and 67.3%, and anastomotic leak rates of 18.4%, 23.5%, and 28.9%, respectively. R0 resection rates were 95.8%, 96.2%, and 96.1%, with pCR rates at 19.9%, 20.3%, and 13.9%. Multivariate logistic regression analysis showed that compared with the interval ≤16 weeks, the longer total preoperative intervals were not significantly associated with the risk of 30-day mortality, overall complications, and anastomotic leakage (*P*>0.05), but the total interval >25 weeks was independently associated with higher ypT stage (OR: 1.58, 95% CI: 1.11–2.23, *P*=0.010) and lower pCR rate (OR: 0.57, 95% CI: 0.37–0.89, *P*=0.013) (Table 4).

**TABLE 4:** Comparison of the Effects of Different Total Preoperative Intervals on Short-term Outcomes and Pathologic Results

Total preoperative interval	Univariate OR (95% CI)	*P*	Adjusted OR (95% CI)	*P*
30-day mortality (5052)				
≤16 wk (719)	Reference		Reference	
16–25 wk (3780)	1.328 (0.768, 2.295)	0.310	0.940 (0.489, 1.805)	0.852
>25 wk (553)	2.787 (1.489, 5.216)	**0.001**	1.794 (0.691, 4.657)	0.230
Overall complications (5052)				
≤16 wk (719)	Reference		Reference	
16–25 wk (3780)	1.268 (1.079, 1.489)	**0.004**	1.019 (0.833, 1.247)	0.855
>25 wk (553)	1.676 (1.331, 2.111)	**<0.001**	0.984 (0.697, 1.390)	0.927
Anastomotic leakage (3877)				
≤16 wk (609)	Reference		Reference	
16–25 wk (2906)	1.366 (1.094, 1.706)	**0.006**	1.045 (0.795, 1.373)	0.753
>25 wk (342)	1.808 (1.325, 2.467)	**<0.001**	1.203 (0.761, 1.903)	0.428
Non-R0 resection (4648)				
≤16 wk (692)	Reference		Reference	
16–25 wk (3497)	0.904 (0.600, 1.362)	0.629	0.847 (0.508, 1.412)	0.524
>25 wk (459)	0.933 (0.512, 1.701)	0.821	1.388 (0.542, 3.552)	0.494
Higher ypT stage (T0-2 vs T3-4) (4883)				
≤16 wk (692)	Reference		Reference	
16–25 wk (3653)	1.068 (0.904, 1.263)	0.439	1.040 (0.842, 1.284)	0.714
>25 wk (538)	1.466 (1.166, 1.843)	**0.001**	1.575 (1.112, 2.230)	**0.010**
Higher ypN stage (N0 vs N+) (4895)				
≤16 wk (693)	Reference		Reference	
16–25 wk (3664)	0.947 (0.802, 1.117)	0.516	0.961 (0.779, 1.184)	0.708
>25 wk (538)	0.844 (0.669, 1.064)	0.151	1.042 (0.734, 1.480)	0.818
pCR (4887)				
≤16 wk (693)	Reference		Reference	
16–25 wk (3656)	1.026 (0.837, 1.257)	0.804	1.028 (0.797, 1.327)	0.830
>25 wk (538)	0.654 (0.481, 0.889)	**0.007**	0.571 (0.367, 0.889)	**0.013**

Bold values are statistical significance of P< 0.05.

The adjusted variables for 30-day mortality, overall complications, and anastomotic leakage included sex, age, Charlson score, history of malignancy, history of thoracic or abdominal surgery, BMI, weight loss, year at diagnosis, histology, tumor location, cT stage, cN stage, ASA, type of resection, diagnosis-to-nCRT interval, nCRT-to-surgery interval, and hospital volume. The adjusted variables for non-R0 resection, higher ypT stage, higher ypN stage, and pCR included history of malignancy, year at diagnosis, histology, tumor location, cT stage, cN stage, diagnosis-to-nCRT interval, nCRT-to-surgery interval, and hospital volume.

BMI indicates body mass index.

## DISCUSSION

This Dutch population-based study showed that none of the preoperative time intervals were related to 30-day mortality, but prolonged diagnosis-to-nCRT and nCRT-to-surgery intervals were associated with more overall complications. In addition, the prolonged nCRT-to-surgery interval was also independently associated with higher risk of anastomotic leakage and lower risk of ypN+. It is worth noting that longer total preoperative interval was associated with higher ypT stage and lower pCR rates.

The analysis of clinical patient characteristics in this study revealed significant correlations among diagnosis-to-nCRT interval, nCRT-to-surgery interval, and the total preoperative interval. The results also demonstrated that the 3 preoperative intervals were independently associated with short-term postoperative outcomes or pathologic results. As previous studies only included a single category preoperative interval, our study results present a more comprehensive impact of all aspects of the preoperative time intervals.

The analysis of the diagnosis-to-nCRT interval showed that a prolonged interval was associated with a higher risk of postoperative overall complications, which may be attributed to selection bias. The results of this study also suggested that patients with longer diagnosis-to-nCRT intervals had worse clinical characteristics and may have needed a longer time to become physically fit for treatment or required extra pretreatment investigations. At present, there is still lack of reliable evidence to prove whether there is a causal relationship between longer diagnosis-to-nCRT interval and worse short-term outcomes. However, multiple studies have indicated that cancer patients endure a significantly increased risk of anxiety and depression and experience a decline in quality of life during the waiting period for treatment.^35–37^

The results of the present study also suggested that a longer nCRT-to-surgery interval was associated with worse short-term outcomes, including higher risk of overall complications and anastomotic leakage. This is consistent with the results of multiple published studies.^27,38–40^ These findings may also be attributed to selection bias, as patients with poorer physical fitness after NAT require longer recovery time before surgery. However, it cannot be ruled out that the worse short-term postoperative outcomes were caused by longer nCRT-to-surgery interval. A longer interval after nCRT can induce more tissue fibrosis, which might increase the difficulty of surgery and thereby the risk of postoperative adverse events.^39^ The results of the NeoRes II trial on patients with esophageal cancer showed that the 10 to 12 weeks nCRT-to-surgery interval had more overall complications and anastomotic leakage than that of 4 to 6 weeks, which was consistent with the results of this study, although it did not reach a significant level.^41^ An international multicenter study also indicated a significant correlation between extended nCRT-to-surgery interval and higher 90-day mortality in patients with esophageal cancer.^42^ Furthermore, a previous randomized controlled trial based on patients with rectal cancer showed that the longer nCRT-to-surgery interval (11 weeks) was associated with higher postoperative morbidity and worse quality of the mesorectal resection than 7 weeks.^43^

The analysis of pathologic results showed that patients with longer nCRT-to-surgery intervals were associated with lower ypN stage, and although their rate of pCR increased, it did not reach significance. As for whether longer nCRT-to-surgery interval can lead to better pathologic results, primarily a higher pCR rate, currently published research has not reached a consistent conclusion.^24–26,28,39,44^ However, almost none of these studies have shown a correlation between extended nCRT-to-surgery interval and improved survival.^24–26,28,39,44,45^ The reason may be that both patients with good response and patients with poor response were included in these studies, and extended nCRT-to-surgery interval may have opposite effects on the pathologic results and survival of these 2 different populations, respectively. For patients with good response to nCRT, extending the interval within a certain range may increase the pCR rate and the level of downstaging, while for patients with poor response to nCRT, extending the interval may even lead to disease progression and worse survival. The recently updated results of NeoRes II trial also support this hypothesis to some extent. Among patients with esophageal cancer with tumor regression grade 4 (>50% remaining tumor cells), patients with 10 to 12 weeks nCRT-to-surgery interval had significantly worse OS compared with patients with a nCRT-to-surgery interval of 4 to 6 weeks. However, the difference in survival between the 2 intervals was not observed in the patients with tumor regression grade 1-3 (histologic complete response; 1%–10% remaining tumor cells; >10%–50% remaining tumor cells).^46^ Furthermore, a multicenter retrospective cohort study based on locally advanced rectal cancer patients with poor response to nCRT has suggested that a longer nCRT-to-surgery interval was associated with poorer OS and disease-free survival.^47^ Even among patients with esophageal cancer who respond well to nCRT, it is still unclear whether they can obtain survival benefits from a longer nCRT-to-surgery interval, although they may obtain better pathologic results after a longer interval. Not all cancer cells that die from nCRT will disintegrate in the short term. Hence, the extension of the nCRT-to-surgery interval may not increase the number of cancer cells that are killed, but only the number of detected cancer cells that are killed, and may increase the risk of proliferation and metastasis of residual cancer cells.^48^ It has become a basic consensus that pCR is associated with better survival, but multiple studies have failed to show that the increase of pCR rate does actually lead to improved survival.^49^ It may be too early to decide to extend the nCRT-to-surgery interval at present.

The results on total preoperative time interval showed that the interval of >25 weeks was associated with an increased risk of higher ypT stage and lower risk of pCR compared with the interval of ≤16 weeks. This implies that a prolonged total preoperative time interval might have led to disease progression and impaired the pCR rate after nCRT. Theoretically, excessive prolongation of either the diagnosis-to-nCRT and/or the nCRT-to-surgery interval may induce preoperative disease progression. At present, there is no evidence that a longer diagnosis-to-NAT interval leads to disease progression or worse survival in patients with esophageal cancer, which may be due to the limited delay before NAT, and the unadjusted impact of the NAT-to-surgery interval could also be a contributing factor.^14,21^ However, as mentioned earlier, multiple studies have shown that longer nCRT-to-surgery interval was associated with poorer survival, probably due to disease progression.^42,46,47^

Based on current evidence, it seems advantageous to shorten the diagnosis-to-nCRT interval as much as possible for patients with esophageal cancer receiving nCRT plus surgery. This could not only reduce the risk of disease progression, and improve quality of life, but also reduce the potential negative impact of diagnosis-to-nCRT interval on short-term postoperative outcomes. For the nCRT-to-surgery interval, the adverse impact of prolonged intervals on the survival of patients with poor response to nCRT has been demonstrated.^46^ As for whether patients with a good response can benefit from longer intervals, further high-quality research is needed.

This study contains the following limitations. First, although using prospectively collected data, this study is a retrospective study and involves selection bias. Second, DUCA has a limited follow-up of only 30 days after surgery and does not collect quality of life, 90-day mortality, and long-term survival data.

## CONCLUSIONS

In patients with esophageal cancer undergoing nCRT and esophagectomy, prolonged preoperative time intervals may lead to higher morbidity and disease progression, and the causal relationship requires further confirmation.

## Supplementary Material

## DISCUSSANT

### Stefan P. Mönig (Geneva, Switzerland)

I would like to thank the ESA for the opportunity to review this paper. Multimodel treatment, incorporating nCRT or chemotherapy alone alongside surgery, has become the standard care in locally advanced esophageal cancer. The time interval from diagnosis to treatment is a crucial indicator for evaluating the quality of cancer care, and the optimal time interval between neoadjuvant treatment and surgery remains a topic of debate. The authors present results from a retrospective analysis using prospective data from the DUCA national auditing registry. They conclude that prolonged preoperative time intervals may lead to higher mobility and disease progression. However, I have some concerns regarding the paper and the methodology used. The most significant limitation of this retrospective data collection is the lack of long-term survival data, including hospital mortality.

I have the following questions: First, the chosen time intervals seem somehow arbitrary, and it appears that the patients in the longer time intervals with higher morbidity are more deconditioned because of higher ASA scores, older age, and more weight loss.

Second, how can you explain the very long waiting time between diagnosis and treatment of 5 to 8 weeks in 42% of the cases in your country, given the centralized treatment of esophageal cancer?

Finally, is there any explication of the very high anastomotic leakage rate compared with published benchmark data in all time intervals between 22.6 and 26.7%?

### Response From Sheraz Markar (London, UK)

The first point to say regarding the chosen time intervals, we did a Cox regression analysis to determine whether there was a maximum inflection point in the data, which there wasn’t. So, then we chose the time intervals based on clinically relevant timing intervals. The first being diagnosis to induction to chemoradiotherapy which is the Dutch national cancer guidelines that I spoke about during the presentation and the second is based on the CROSS data and the data from NEORES2, and the follow-up CROSS trial data which suggested that patients who waited more than 12 weeks after surgery had an adverse outcome.

The second question about deconditioning of patients is a very valid one. So obviously within a retrospective study, you are able to regress for all of the factors that you have highlighted within your presentation; however, some variables are unmeasurable within these data sets which may account for some of the factors that we see with some of the patients who wait a bit longer and maybe deconditioned during chemoradiotherapy, and this is a valid concern.

Additionally, your comment about the 42% of patients waiting 5 to 8 weeks in the centralized service, is reflective of the centralization. Patients do come from far away for their treatment within Holland like they do in the UK. In the UK, I must admit we are a little bit tighter on our timelines for treatment, but I think this reflects changes in practice over time through a period of centralization within a country.

Finally, your question on the anastomotic leak, it is hard for me to comment on this. As a UK-based surgeon, I feel that the anastomotic rate with the study is high, however, this is not unusual for Dutch data as this is presented many times in our international meetings. If I were Dutch, I probably would say that the fact that they have very robust collection sets for the data and therefore this is something that they search for the leak a little bit more aggressively than we do in other countries, but that said, I am not Dutch.
