# Supplementary material for: Impact of Preoperative Time Intervals for Neoadjuvant Chemoradiotherapy on Short-term Postoperative Outcomes of Esophageal Cancer Surgery: A Population-based Study Using the Dutch Upper Gastrointestinal Cancer Audit (DUCA) Data
Source: Ann Surg. 2024 Aug 8;280(5):808–16. doi: 10.1097/SLA.0000000000006476 (PMC11446532; doi:10.1097/SLA.0000000000006476)
Supplement: Supplementary file 1 [file sla-280-808-s001.docx]

**Table 1.** The clinical characteristics of esophageal cancer patients with different diagnosis-to-nCRT intervals.

| **Variables** | **All (5052)** | **≤5 weeks (2474)** | **5-8 weeks (2130)** | **8-12 weeks (448)** | **p-value** |
| --- | --- | --- | --- | --- | --- |
| Age |  |  |  |  | **<0.001** |
| 18-64 | 2203(43.6%) | 1132(45.8%) | 910(42.7%) | 161(35.9%) |  |
| 65-80 | 2733(54.1%) | 1294(52.3%) | 1171(55.0%) | 268(59.8%) |  |
| ≥80 | 116(25.6) | 48(1.9%) | 49(2.3%) | 19(4.2%) |  |
| BMI |  |  |  |  | 0.374 |
| <20 | 308(6.1%) | 155(6.3%) | 123(5.8%) | 30(6.7%) |  |
| 20-25 | 1874(37.1%) | 911(36.8%) | 804(37.7%) | 159(35.5%) |  |
| 25-30 | 2023(40.0%) | 1017(41.1%) | 836(39.2%) | 170(37.9%) |  |
| ≥30 | 822(16.3%) | 382(15.4%) | 355(16.7%) | 85(19.0%) |  |
| unknown | 25(0.5%) | 9(0.4%) | 12(0.6) | 4(0.9%) |  |
| weight loss |  |  |  |  | 0.164 |
| <5 kg | 2525(50.0%) | 1255(50.7%) | 1063(49.9%) | 207(46.2%) |  |
| ≥5 kg | 2133(42.2%) | 1041(42.1%) | 897(42.1%) | 195(43.5%) |  |
| unknown | 394(7.8%) | 178(7.2) | 170(8.0%) | 46(10.3%) |  |
| Sex |  |  |  |  | 0.827 |
| male | 3997(79.1%) | 1950(78.8%) | 1694(79.5%) | 353(78.8%) |  |
| female | 1055(20.9%) | 524(21.2%) | 436(20.5%) | 95(21.2%) |  |
| Charlson score |  |  |  |  | **0.007** |
| 0 | 2449(48.5%) | 1213(49.0%) | 1049(49.2%) | 187(41.7%) |  |
| 1 | 1290(25.5%) | 637(25.7%) | 540(25.4%) | 113(25.2%) |  |
| 2+ | 1313(26.0) | 624(25.2%) | 541(25.4%) | 148(33.0%) |  |
| History of malignancy |  |  |  |  | **0.018** |
| no | 4345(86.0%) | 2130(86.1%) | 1949(86.8%) | 366(81.7%) |  |
| yes | 707(14.0%) | 344(13.9%) | 281(13.2%) | 82(18.3%) |  |
| History of thoracic and abdominal surgery |  |  |  |  | 0.143 |
| no | 3640(72.1%) | 1802(72.8%) | 1537(72.2%) | 301(67.2%) |  |
| yes | 1406(27.8%) | 669(27.0%) | 590(27.7%) | 147(32.8%) |  |
| unknown | 6(0.1%) | 3(0.1%) | 3(0.1%) | 0(0%) |  |
| Referral |  |  |  |  | **<0.001** |
| no | 1166(23.1%) | 621(25.1%) | 466(21.9%) | 79(17.6%) |  |
| yes | 3529(69.9%) | 1699(68.7%) | 1505(70.7%) | 325(72.5%) |  |
| unknown | 357(7.1%) | 154(6.2%) | 159(7.5%) | 44(9.8%) |  |
| Year of diagnosis |  |  |  |  | **<0.001** |
| 2010-2013 | 1233(24.4%) | 492(19.9%) | 605(28.4%) | 136(30.4%) |  |
| 2014-2017 | 2223(44.0%) | 1121(45.3%) | 932(43.8%) | 170(37.9%) |  |
| 2018-2021 | 1596(31.6%) | 861(34.8%) | 593(27.8%) | 142(31.7%) |  |
| Histology |  |  |  |  | 0.628 |
| adenocarcinoma | 4007(79.3%) | 1957(79.1%) | 1702(79.9%) | 348(77.7%) |  |
| squamous cell carcinoma | 921(18.2%) | 453(18.3%) | 384(18.0%) | 84(18.8%) |  |
| other | 79(1.6%) | 42(1.7%) | 27(1.3%) | 10(2.2%) |  |
| unknown | 45(0.9%) | 22(0.9%) | 17(0.8%) | 6(1.3%) |  |
| Tumor location |  |  |  |  | 0.503 |
| intrathoracal proximal esophagus | 51(1.0%) | 23(0.9%) | 26(1.2%) | 2(0.4%) |  |
| intrathoracal middle esophagus | 579(11.5%) | 263(10.6%) | 259(12.2%) | 57(12.7%) |  |
| intrathoracal distal esophagus | 3585(71.0%) | 1773(71.7%) | 1490(70.0%) | 322(71.9%) |  |
| esophagogastric junction | 832(16.5%) | 412(16.7%) | 353(16.6%) | 67(15.0%) |  |
| unknown | 5(0.1%) | 3(0.1%) | 2(0.1%) | 0(0%) |  |
| cT stage |  |  |  |  | **0.005** |
| T1 | 36(0.7%) | 16(0.6%) | 17(0.8%) | 3(0.7%) |  |
| T2 | 991(19.6%) | 443(17.9%) | 438(20.6%) | 110(24.6%) |  |
| T3 | 3909(77.4%) | 1969(79.6%) | 1618(76.0%) | 322(71.9%) |  |
| T4 | 116(2.3%) | 46(1.9%) | 57(2.7%) | 13(2.9%) |  |
| cN stage |  |  |  |  | 0.898 |
| N0 | 1732(34.3%) | 844(34.1%) | 734(34.5%) | 154(34.4%) |  |
| N1 | 2119(41.9%) | 1044(42.2%) | 894(42.0%) | 181(40.4%) |  |
| N2 | 983(19.5%) | 484(19.6%) | 408(19.2%) | 91(20.3%) |  |
| N3 | 134(2.7%) | 68(2.7%) | 54(2.5%) | 12(2.7%) |  |
| unknown | 84(1.7%) | 34(1.4%) | 40(1.9%) | 10(2.2%) |  |
| ASA score |  |  |  |  | **0.015** |
| 1 | 682(13.5%) | 350(14.1%) | 278(13.1%) | 54(12.1%) |  |
| 2 | 3096(61.3%) | 1540(62.2%) | 1290(60.6%) | 266(59.4%) |  |
| 3 | 1216(24.1%) | 547(22.1%) | 545(25.6%) | 124(27.7%) |  |
| 4 | 30(0.6%) | 16(0.6%) | 11(0.5%) | 3(0.7%) |  |
| unknown | 28(0.6%) | 21(0.8%) | 6(0.3%) | 1(0.2%) |  |
| Type of resection |  |  |  |  | 0.723 |
| transhiatal esophagectomy | 1075(21.3%) | 536(21.7%) | 449(21.1%) | 90(20.1%) |  |
| transthoracic esophagectomy | 3977(78.7%) | 1938(78.3%) | 1681(78.9%) | 358(79.9%) |  |
| nCRT-to-surgery interval |  |  |  |  | **<0.001** |
| 5-11 weeks | 667(13.2%) | 268(10.8%) | 317(14.9%) | 82(18.3%) |  |
| 11-17 weeks | 3424(67.8%) | 1736(70.2%) | 1429(67.1%) | 259(57.8%) |  |
| >17 weeks | 961(19.0%) | 470(19.0%) | 384(18.0%) | 107(23.9%) |  |
| Total interval (diagnosis to surgery) |  |  |  |  | **<0.001** |
| ≤16 weeks | 719(14.2%) | 611(24.7%) | 105(4.9%) | 3(0.7%) |  |
| 16-25 weeks | 3780(74.8%) | 1696(68.6%) | 1786(83.8%) | 298(66.5%) |  |
| >25 weeks | 553(10.9%) | 167(6.8%) | 239(11.2%) | 147(32.8%) |  |
| Hospital volume |  |  |  |  | 0.305 |
| <40 | 2061(40.8%) | 1032(41.7%) | 858(40.3%) | 171(38.2%) |  |
| ≥40 | 2991(59.2%) | 1442(58.3%) | 1272(59.7%) | 277(61.8%) |  |

**Table 2.** The clinical characteristics of esophageal cancer patients with different nCRT-to-surgery intervals.

| **Variables** | **All (5052)** | **5-11 weeks (667)** | **11-17 weeks (3424)** | **>17 weeks (961)** | **p-value** |
| --- | --- | --- | --- | --- | --- |
| Age |  |  |  |  | **0.004** |
| 18-64 | 2203(43.6%) | 316(47.4%) | 1518(44.3%) | 369(38.4%) |  |
| 65-80 | 2733(54.1%) | 339(50.8%) | 1827(53.4%) | 567(59.0%) |  |
| ≥80 | 116(2.3) | 12(1.8) | 79(2.3%) | 25(2.6%) |  |
| BMI |  |  |  |  | 0.467 |
| <20 | 308(6.1%) | 40(6.0%) | 210(6.1%) | 58(6.0%) |  |
| 20-25 | 1874(37.1%) | 259(38.8%) | 1291(37.7%) | 324(33.7%) |  |
| 25-30 | 2023(40.0%) | 262(39.3%) | 1361(39.7%) | 400(41.6%) |  |
| ≥30 | 822(16.3%) | 103(15.4%) | 544(15.9%) | 175(18.2%) |  |
| unknown | 25(0.5%) | 3(0.4%) | 18(0.5) | 4(0.4%) |  |
| weight loss |  |  |  |  | **<0.001** |
| <5 kg | 2525(50.0%) | 336(50.4%) | 1706(49.8%) | 483(50.3%) |  |
| ≥5 kg | 2133(42.2%) | 248(37.2%) | 1453(42.4%) | 432(45.0%) |  |
| unknown | 394(7.8%) | 83(12.4) | 265(7.7%) | 46(4.8%) |  |
| Sex |  |  |  |  | 0.656 |
| male | 3997(79.1%) | 521(78.1%) | 2721(79.5%) | 755(78.6%) |  |
| female | 1055(20.9%) | 146(21.9%) | 703(20.5%) | 206(21.4%) |  |
| Charlson score |  |  |  |  | **<0.001** |
| 0 | 2449(48.5%) | 364(54.6%) | 1671(48.8%) | 414(43.1%) |  |
| 1 | 1290(25.5%) | 155(23.2%) | 889(26.0%) | 246(25.6%) |  |
| 2+ | 1313(26.0) | 148(22.2%) | 864(25.2%) | 301(31.3%) |  |
| History of malignancy |  |  |  |  | 0.4 |
| no | 4345(86.0%) | 579(86.8%) | 2952(86.2%) | 814(84.7%) |  |
| yes | 707(14.0%) | 88(13.2%) | 472(13.8%) | 147(15.3%) |  |
| History of thoracic and abdominal surgery |  |  |  |  | 0.177 |
| no | 3640(72.1%) | 484(72.6%) | 2486(72.6%) | 670(69.7%) |  |
| yes | 1406(27.8%) | 183(27.4%) | 932(27.2%) | 291(30.3%) |  |
| unknown | 6(0.1%) | 0(0%) | 6(0.2%) | 0(0%) |  |
| Referral |  |  |  |  | **<0.001** |
| no | 1166(23.1%) | 170(25.5%) | 814(23.8%) | 182(18.9%) |  |
| yes | 3529(69.9%) | 398(59.7%) | 2389(69.8%) | 742(77.2%) |  |
| unknown | 357(7.1%) | 99(14.8%) | 221(6.5%) | 37(3.9%) |  |
| Year of diagnosis |  |  |  |  | **<0.001** |
| 2010-2013 | 1233(24.4%) | 307(46.0%) | 840(24.5%) | 86(8.9%) |  |
| 2014-2017 | 2223(44.0%) | 298(44.7%) | 1595(46.6%) | 330(34.3%) |  |
| 2018-2021 | 1596(31.6%) | 62(9.3%) | 989(28.9%) | 545(56.7%) |  |
| Histology |  |  |  |  | 0.495 |
| adenocarcinoma | 4007(79.3%) | 517(77.5%) | 2730(79.7%) | 760(79.1%) |  |
| squamous cell carcinoma | 921(18.2%) | 138(20.7%) | 609(17.8%) | 174(18.1%) |  |
| other | 79(1.6%) | 7(1.0%) | 53(1.5%) | 19(2.0%) |  |
| unknown | 45(0.9%) | 5(0.7%) | 32(0.9%) | 8(0.8%) |  |
| Tumor location |  |  |  |  | 0.123 |
| intrathoracal proximal esophagus | 51(1.0%) | 3(0.4%) | 36(1.1%) | 12(1.2%) |  |
| intrathoracal middle esophagus | 579(11.5%) | 80(12.0%) | 386(11.3%) | 113(11.8%) |  |
| intrathoracal distal esophagus | 3585(71.0%) | 471(70.6%) | 2406(70.3%) | 708(73.7%) |  |
| esophagogastric junction | 832(16.5%) | 113(16.9%) | 592(17.3%) | 127(13.2%) |  |
| unknown | 5(0.1%) | 0(0%) | 4(0.1%) | 1(0.1%) |  |
| cT stage |  |  |  |  | 0.254 |
| T1 | 36(0.7%) | 5(0.7%) | 28(0.8%) | 3(0.3%) |  |
| T2 | 991(19.6%) | 137(20.5%) | 675(19.7%) | 179(18.6%) |  |
| T3 | 3909(77.4%) | 516(77.4%) | 2642(77.2%) | 751(78.1%) |  |
| T4 | 116(2.3%) | 9(1.3%) | 79(2.3%) | 28(2.9%) |  |
| cN stage |  |  |  |  | **0.047** |
| N0 | 1732(34.3%) | 217(32.5%) | 1183(34.6%) | 332(34.5%) |  |
| N1 | 2119(41.9%) | 291(43.6%) | 1442(42.1%) | 386(40.2%) |  |
| N2 | 983(19.5%) | 123(18.4%) | 654(19.1%) | 206(21.4%) |  |
| N3 | 134(2.7%) | 15(2.2%) | 93(2.7%) | 26(2.7%) |  |
| unknown | 84(1.7%) | 21(3.1%) | 52(1.5%) | 11(1.1%) |  |
| ASA score |  |  |  |  | **<0.001** |
| 1 | 682(13.5%) | 123(18.4%) | 470(13.7%) | 89(9.3%) |  |
| 2 | 3096(61.3%) | 420(63.0%) | 2107(61.5%) | 569(59.2%) |  |
| 3 | 1216(24.1%) | 120(18.0%) | 811(23.7%) | 285(29.7%) |  |
| 4 | 30(0.6%) | 2(0.3%) | 13(0.4%) | 15(1.6%) |  |
| unknown | 28(0.6%) | 2(0.3%) | 23(0.7%) | 3(0.3%) |  |
| Type of resection |  |  |  |  | **<0.001** |
| transhiatal esophagectomy | 1075(21.3%) | 239(35.8%) | 698(20.4%) | 138(14.4%) |  |
| transthoracic esophagectomy | 3977(78.7%) | 428(64.2%) | 2726(79.6%) | 823(85.6%) |  |
| Diagnosis-to-nCRT interval |  |  |  |  | **<0.001** |
| ≤5 weeks | 2474(49.0%) | 268(40.2%) | 1736(50.7%) | 470(48.9%) |  |
| 5-8 weeks | 2130(42.2%) | 317(47.5%) | 1429(41.7%) | 384(40.0%) |  |
| 8-12 weeks | 448(8.9%) | 82(12.3%) | 259(7.6%) | 107(11.1%) |  |
| Total interval(diagnosis to surgery) |  |  |  |  | **<0.001** |
| ≤16 weeks | 719(14.2%) | 376(56.4%) | 343(10.0%) | 0(0%) |  |
| 16-25 weeks | 3780(74.8%) | 291(43.6%) | 3041(88.8%) | 448(46.6%) |  |
| >25 weeks | 553(10.9%) | 0(0%) | 40(1.2%) | 513(53.4%) |  |
| Hospital volume |  |  |  |  | **<0.001** |
| <40 | 2061(40.8%) | 345(51.7%) | 1443(42.1%) | 273(28.4%) |  |
| ≥40 | 2991(59.2%) | 322(48.3%) | 1981(57.9%) | 688(71.6%) |  |

**Table 3.** The clinical characteristics of esophageal cancer patients with different total preoperative intervals.

| **Variables** | **All (5052)** | **≤16 weeks (719)** | **16-25weeks (3780)** | **>25 weeks (553)** | **p-value** |
| --- | --- | --- | --- | --- | --- |
| Age |  |  |  |  | **<0.001** |
| 18-64 | 2203(43.6%) | 355(49.4%) | 1651(43.7%) | 197(35.6%) |  |
| 65-80 | 2733(54.1%) | 355(49.4%) | 2042(54.0%) | 336(60.8%) |  |
| ≥80 | 116(25.6%) | 9(1.3%) | 87(2.3%) | 20(3.6%) |  |
| BMI |  |  |  |  | 0.172 |
| <20 | 308(6.1%) | 46(6.4%) | 230(6.1%) | 32(5.8%) |  |
| 20-25 | 1874(37.1%) | 287(39.9%) | 1404(37.1%) | 183(33.1%) |  |
| 25-30 | 2023(40.0%) | 285(39.6%) | 1511(40.0%) | 227(41.0%) |  |
| ≥30 | 822(16.3%) | 99(13.8%) | 616(19.3%) | 107(19.3%) |  |
| unknown | 25(0.3%) | 2(0.3%) | 19(0.5) | 4(0.7%) |  |
| weight loss |  |  |  |  | **<0.001** |
| <5 kg | 2525(50.0%) | 345(48.0%) | 1897(50.2%) | 283(51.2%) |  |
| ≥5 kg | 2133(42.2%) | 288(40.1%) | 1602(42.4%) | 243(43.9%) |  |
| unknown | 394(7.8%) | 86(12.0) | 281(7.4%) | 27(4.9%) |  |
| Sex |  |  |  |  | 0.548 |
| male | 3997(79.1%) | 558(77.6%) | 3002(79.4%) | 437(79.0%) |  |
| female | 1055(20.9%) | 161(22.4%) | 778(20.6%) | 116(21.0%) |  |
| Charlson score |  |  |  |  | **<0.001** |
| 0 | 2449(48.5%) | 396(55.1%) | 1830(48.4%) | 223(40.3%) |  |
| 1 | 1290(25.5%) | 169(23.5%) | 978(25.9%) | 143(25.9%) |  |
| 2+ | 1313(26.0) | 154(21.4%) | 972(25.7%) | 187(33.8%) |  |
| History of malignancy |  |  |  |  | **0.037** |
| no | 4345(86.0%) | 624(86.8%) | 3265(86.4%) | 456(82.5%) |  |
| yes | 707(14.0%) | 95(13.2%) | 515(13.6%) | 97(17.5%) |  |
| History of thoracic and abdominal surgery |  |  |  |  | **0.038** |
| no | 3640(72.1%) | 513(71.3%) | 2755(72.9%) | 372(67.3%) |  |
| yes | 1406(27.8%) | 206(28.7%) | 1019(27.0%) | 181(32.7%) |  |
| unknown | 6(0.1%) | 0(0%) | 6(0.2%) | 0(0%) |  |
| Referral |  |  |  |  | **<0.001** |
| no | 1166(23.1%) | 212(29.5%) | 861(22.8%) | 93(16.8%) |  |
| yes | 3529(69.9%) | 435(60.5%) | 2654(70.2%) | 440(79.6%) |  |
| unknown | 357(7.1%) | 72(10.0%) | 265(7.0%) | 20(3.6%) |  |
| Year of diagnosis |  |  |  |  | **<0.001** |
| 2010-2013 | 1233(24.4%) | 267(37.1%) | 918(24.3%) | 48(8.7%) |  |
| 2014-2017 | 2223(44.0%) | 347(48.3%) | 1706(45.1%) | 170(30.7%) |  |
| 2018-2021 | 1596(31.6%) | 105(14.6%) | 1156(30.6%) | 335(60.6%) |  |
| Histology |  |  |  |  | 0.558 |
| adenocarcinoma | 4007(79.3%) | 568(79.0%) | 3001(79.4%) | 438(79.2%) |  |
| squamous cell carcinoma | 921(18.2%) | 139(19.3%) | 683(18.1%) | 99(17.9%) |  |
| other | 79(1.6%) | 9(1.3%) | 58(1.5%) | 12(2.2%) |  |
| unknown | 45(0.9%) | 3(0.4%) | 38(1.0%) | 4(0.7%) |  |
| Tumor location |  |  |  |  | 0.123 |
| intrathoracal proximal esophagus | 51(1.0%) | 7(1.0%) | 36(1.0%) | 8(1.4%) |  |
| intrathoracal middle esophagus | 579(11.5%) | 78(10.8%) | 437(11.6%) | 64(11.6%) |  |
| intrathoracal distal esophagus | 3585(71.0%) | 506(70.4%) | 2664(70.5%) | 415(75.0%) |  |
| esophagogastric junction | 832(16.5%) | 128(17.8%) | 639(16.9%) | 65(11.8%) |  |
| unknown | 5(0.1%) | 0(0%) | 4(0.1%) | 1(0.2%) |  |
| cT stage |  |  |  |  | 0.753 |
| T1 | 36(0.7%) | 7(1.0%) | 27(0.7%) | 2(0.4%) |  |
| T2 | 991(19.6%) | 144(20.0%) | 731(19.3%) | 116(21.0%) |  |
| T3 | 3909(77.4%) | 555(77.2%) | 2931(77.5%) | 423(76.5%) |  |
| T4 | 116(2.3%) | 13(1.8%) | 91(2.4%) | 12(2.2%) |  |
| cN stage |  |  |  |  | 0.571 |
| N0 | 1732(34.3%) | 233(32.4%) | 1299(34.4%) | 200(36.2%) |  |
| N1 | 2119(41.9%) | 319(44.4%) | 1581(41.8%) | 219(39.6%) |  |
| N2 | 983(19.5%) | 132(18.4%) | 738(19.5%) | 113(20.4%) |  |
| N3 | 134(2.7%) | 18(2.5%) | 102(2.7%) | 14(2.5%) |  |
| unknown | 84(1.7%) | 17(2.4%) | 60(1.6%) | 7(1.3%) |  |
| ASA score |  |  |  |  | **<0.001** |
| 1 | 682(13.5%) | 133(18.5%) | 508(13.4%) | 41(7.4%) |  |
| 2 | 3096(61.3%) | 469(65.2%) | 2301(60.9%) | 326(59.0%) |  |
| 3 | 1216(24.1%) | 112(15.6%) | 928(24.6%) | 176(31.8%) |  |
| 4 | 30(0.6%) | 3(0.4%) | 18(0.5%) | 9(1.6%) |  |
| unknown | 28(0.6%) | 2(0.3%) | 25(0.7%) | 1(0.2%) |  |
| Type of resection |  |  |  |  | **<0.001** |
| transhiatal esophagectomy | 1075(21.3%) | 242(33.7%) | 747(19.8%) | 86(15.6%) |  |
| transthoracic esophagectomy | 3977(78.7%) | 477(66.3%) | 3033(80.2%) | 467(84.4%) |  |
| Diagnosis-to-nCRT interval |  |  |  |  | **<0.001** |
| ≤5 weeks | 2474(49.0%) | 611(85.0%) | 1696(44.9%) | 167(30.2%) |  |
| 5-8 weeks | 2130(42.2%) | 105(14.6%) | 1786(47.2%) | 239(43.2%) |  |
| 8-12 weeks | 448(8.9%) | 3(0.4%) | 298(7.9%) | 147(26.6%) |  |
| nCRT-to-surgery interval |  |  |  |  | **<0.001** |
| 5-11 weeks | 667(13.2%) | 376(52.3%) | 291(7.7%) | 0(0%) |  |
| 11-17 weeks | 3424(67.8%) | 343(47.7%) | 3041(80.4%) | 40(7.2%) |  |
| >17 weeks | 961(19.0%) | 0(0%) | 448(11.9%) | 513(92.8%) |  |
| Hospital volume |  |  |  |  | **<0.001** |
| <40 | 2061(40.8%) | 376(52.3%) | 1539(40.7%) | 146(26.4%) |  |
| ≥40 | 2991(59.2%) | 343(47.7%) | 2241(59.3%) | 407(73.6%) |  |
